# Renal function and anemia in relation to short- and long-term prognosis of patients with acute heart failure in the period 1985-2008: A clinical cohort study

**DOI:** 10.1371/journal.pone.0201714

**Published:** 2018-08-07

**Authors:** Jan C. van den Berge, Alina A. Constantinescu, Ron T. van Domburg, Milos Brankovic, Jaap W. Deckers, K. Martijn Akkerhuis

**Affiliations:** Department of Cardiology, Thoraxcenter, Erasmus MC Rotterdam, the Netherlands; Scuola Superiore Sant'Anna, ITALY

## Abstract

**Background:**

Renal dysfunction and anaemia are common in patients with acute heart failure (HF). It is not known whether their combined presence has additive prognostic value. We investigated their prognostic value separately and in combination, on prognosis in acute HF patients. Furthermore, we examined whether the improvement in prognosis was comparable between patients with and without renal dysfunction.

**Methods and results:**

This prospective registry includes 1783 patients admitted to the (Intensive) Coronary Care Unit for acute HF in the period of 1985–2008. The outcome measure was the composite of all-cause mortality, heart transplantation and left ventricular assist device implantation. In patients without renal dysfunction, anemia was associated with worse 30-day outcome (HR 2.91; [95% CI 1.69–5.00]), but not with 10-year outcome (HR 1.13 [95% CI 0.93–1.37]). On the contrary, anemia was found to influence prognosis in patients with renal dysfunction, both at 30 days (HR 1.93 [95% CI 1.33–2.80]) and at 10 years (HR 1.27 [95% CI 1.10–1.47]). Over time, the 10-year survival rate improved in patients with preserved renal function (HR 0.73 [95% CI 0.55–0.97]), but not in patients with renal dysfunction.

**Conclusion:**

The long-term prognosis of acute HF patients with a preserved renal function was found to have improved significantly. However, the prognosis of patients with renal dysfunction did not change. Anemia was a strong prognosticator for short-term outcome in all patients. In patients with renal dysfunction, anemia was also associated with impaired long-term prognosis.

## Introduction

Acute heart failure (HF) is commonly accompanied by various non-cardiovascular comorbidities. Renal dysfunction is among one of the most common although its exact prevalence has varied between studies.[1, 2] Renal dysfunction in acute HF is associated with various adverse outcomes: longer hospital stay, higher re-hospitalization rate, and higher mortality.[1, 2] Of note, the follow-up period in most of these studies is restricted to only 1 year after the initial hospitalization.

In the last decades, an improvement in long-term outcome has been observed among patients with acute HF in several cohorts.[3–5] New therapeutic options and an increased understanding of the pathophysiology of HF are most likely responsible for this trend. Importantly, renal dysfunction is a (relative) contra-indication for some of the new therapeutic modalities[6]. As of yet, it has not been established whether the improvement in prognosis over time of patients with acute HF is modified by the presence of renal dysfunction.

Anemia is another important and common comorbidity in patients with acute HF, with a prevalence up to almost 60%.[7–12] There is conflicting data regarding the prognostic impact of anemia in patients with acute HF.[10–13] Moreover, the combination of HF, renal dysfunction and anemia carries an incremental negative prognostic impact in patients with *chronic* HF.[14] However, the additive prognostic value of anemia in patients with *acute* HF with and without renal dysfunction remains scarce.

Therefore, the aims of the present study were (1) to examine the impact of renal function on short- and long-term prognosis of patients with acute HF, (2) to determine whether the improvement in prognosis of patients with acute HF and renal impairment was comparable to that of patients with normal renal function, and (3) to study the impact of anemia, alone or in combination with renal dysfunction, on prognosis of patients with acute HF.

## Materials and methods

### Patients

This prospective registry was carried out among patients who were admitted with acute HF at the Intensive Coronary Care Unit (ICCU) in our hospital during the period from 1985 until 2008. The study design and inclusion have been described previously.[5] Briefly, consecutive patients aged 18 years and older were included when they were diagnosed with acute HF or cardiogenic shock at admission. Both patients with de novo HF and patients with worsening symptoms of chronic HF were included. Patients could only contribute once to the database, and if patients were admitted more than once with acute HF during the inclusion period, only the first admission was included for analyses.

This was a prospective cohort registry. For analyses, we used completely anonymized data. During the enrolment of the patients, approval from the research ethics committee of the Erasmus MC to conduct this study was not required. At a later stage, the committee confirmed that we did not need their approval to conduct this study. Furthermore, there was no requirement for patients’ informed consent. The study was conducted according to the Declaration of Helsinki.[15]

### Endpoints

The outcome measure was the composite of all-cause mortality, heart transplantation and left ventricular assist device (LVAD) implantation at 30 days, 1 year and 10 years after the initial hospitalization.

Survival status was assessed by using the Municipal Civil Registries in January 2017 and was available for 98% of the included patients. To determine whether patients received an LVAD or underwent heart transplantation, we used prospectively collected data from our hospital information system.

### Variables and definitions

Baseline variables were derived from patient records and discharge letters. We collected the following variables: age, gender, Body Mass Index (BMI), cardiac history, etiology of HF, left ventricular ejection fraction (LVEF) and treatment at the ICCU. Furthermore, the results of the following laboratory tests were collected: sodium (mmol/L), potassium (mmol/L), creatinine (μmol/L), urea (mmol/L) and hemoglobin (mmol/L).

Diabetes mellitus was considered to be present when patients received antidiabetic therapy. The LVEF was classified into the following qualitative categories: good, moderate and poor. If quantitative outcome for the LVEF was used, we applied the following cut-offs: >45%, 30–44% and <30% for good, moderate and poor LVEF, respectively.[5] The etiology of HF was categorized into ischemic cause versus non-ischemic cause of HF. For all laboratory tests, the first measured value during hospitalization was taken into account. The estimated glomerular filtration rate (eGFR) was estimated by using the Modification of Diet in Renal Disease (MDRD) equation for serum creatinine (μmol/L): eGFR = 30849 × serum creatinine ^−1.154^ × age ^−0.203^ × 0.742 (if female) [eGFR in mL/min/1.73 m^2^].[16] In line with the most recent HF guideline of the European Society of Cardiology,[6] renal function was categorized as follows: preserved renal function: eGFR ≥60 mL/min/1.73 m^2^; moderately impaired renal function eGFR 30–59 mL/min/1.73 m^2^; severely impaired renal function eGFR <30 mL/min/1.73 m^2^. We used the definition of the World Health Organization to define anemia: hemoglobin <7.5 mmol/L in women and <8.2 mmol/L in men. Hyponatremia was defined as a serum sodium level ≤135 mmol/L. For the definition of hypo- and hyperkalemia the following cut-off values were applied: serum potassium <3.5 mmol/L and >5.0 mmol/L, respectively.

### Statistical analysis

Categorical variables are presented as frequencies and percentages. The χ^2^ test and the Fisher-Freeman-Halton exact test were used to compare categorical variables. Normally distributed, continuous data are presented as mean values with standard deviation and were compared using the one-way ANOVA. Continuous data that were not normally distributed are presented as median and interquartile range (IQR). The Mann-Whitney U test or the Kruskal-Wallis H test was used to compare these data.

Since data for LVEF and etiology were incomplete for, respectively, 28% and 12% of the patients, multiple imputation was performed by using baseline characteristics as predictors. Pooled means are given for LVEF and etiology.

The Kaplan-Meier method was used for presenting the cumulative event curves and they were compared using the log-rank test. Secondary analyses were carried out among the 30-day event-free survivors. Logistic regression for 30-day outcome and the Cox proportional hazard method for long-term outcome were applied in order to examine the independent association between renal function and the composite endpoint of all-cause mortality, heart transplantation and LVAD implantation, as well as between anemia and the composite endpoint. Adjustments were made for age, gender, history of HF, diabetes, hypertension, etiology of HF, atrial fibrillation at admission, LVEF, renal function and anemia.

All tests were two-tailed and p-values <0.05 were considered statistically significant. Results of logistic regression and the Cox proportional hazard model were reported as odds ratios (ORs) and hazard ratios (HRs), respectively, with their corresponding 95% confidence interval (95% CI). All statistical analyses were carried out using SPSS software (SPSS 21.0, IBM Corp., Armonk, NY, USA).

## Results

### Baseline characteristics

**Fig 1:**
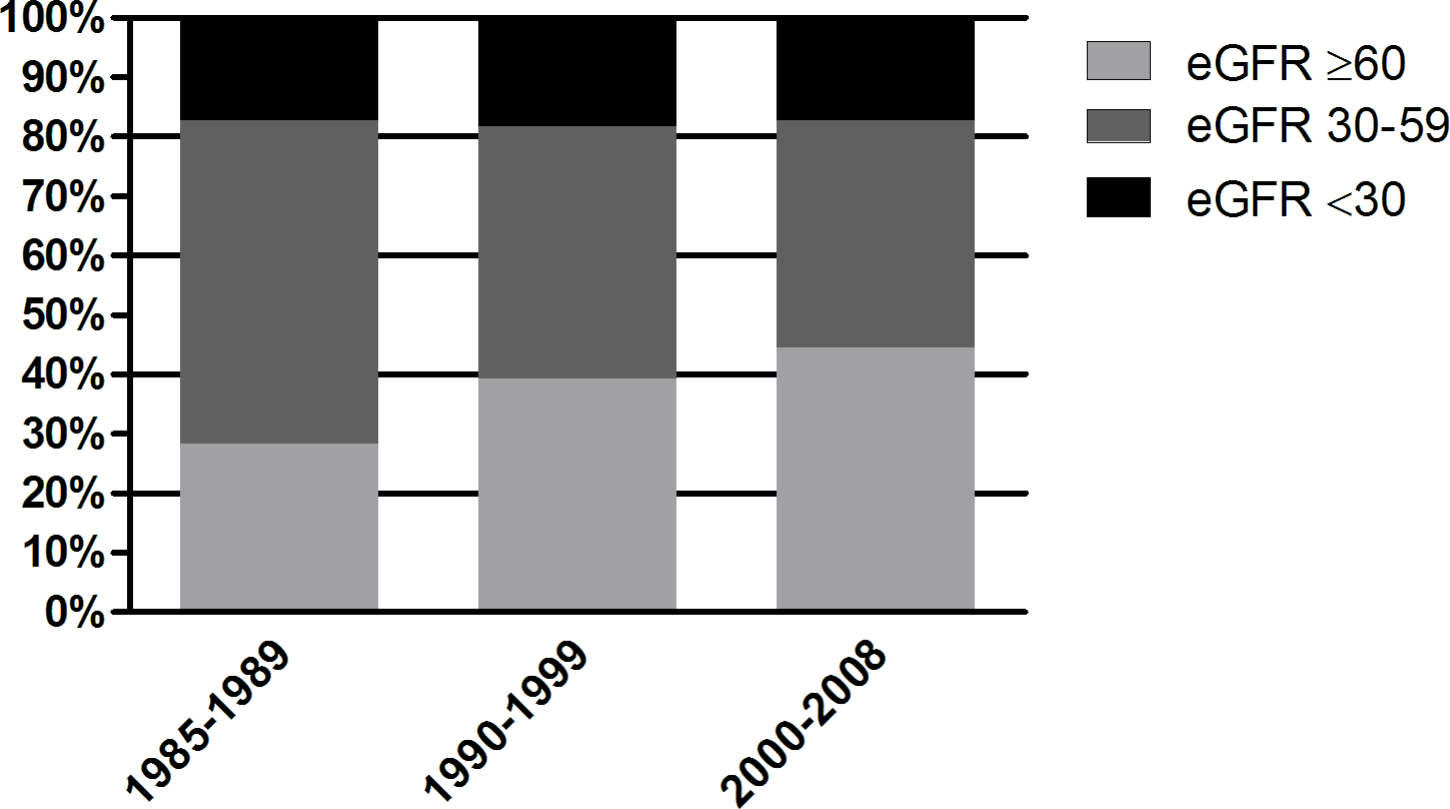
Distribution of the study population according to the renal function and the admission period. eGFR, estimated glomerular filtration rate in mL/min/1.73 m^2^.

In total, 1810 patients were admitted with acute HF in the period 1985–2008. Of these, 1783 (99%) patients had at least one creatinine measurement and they constitute the present study population. Over half of the patients were found to have renal dysfunction, which was severely impaired in 18%. The proportion of patients with severe renal impairment remained stable over time, whereas the number of patients with preserved renal function increased and moderately impaired renal function became less prevalent (p<0.001; Fig 1).

Compared to patients with renal dysfunction, patients with preserved renal function were on average 6 years younger (Table 1). In addition, they less often had prior myocardial infarction and coronary revascularization. With decreasing renal function, the prevalence of prior HF, diabetes and hypertension increased. Hyponatremia was also more common in patients with renal dysfunction, as was anemia.

**Table 1:** Baseline characteristics and therapy according to renal function.

	eGFR ≥60		eGFR 30–59	eGFR <30	p-value*
No. of patients	688 (39%)		778 (44%)	317 (18%)	
*Baseline*					
Age (mean, y)	59.7 ± 16.3		66.1 ± 13.2	65.9 ± 12.9	<0.001
Male	458 (67%)		475 (61%)	201 (63%)	0.09
BMI	25.4 ± 5.2		24.9 ± 4.8	25.0 ± 4.7	0.57
*Medical history*					
Myocardial infarction	237 (34%)		347 (45%)	120 (38%)	<0.001
Coronary revascularization†	124 (18%)		187 (24%)	75 (24%)	0.01
Heart surgery (not CABG)	111 (16%)		87 (11%)	36 (11%)	0.01
Heart transplantation	2 (0.3%)		1 (0.1%)	6 (2%)	0.002
Waiting for heart transplantation	16 (2.3%)		11 (1.4%)	8 (2.5%)	0.33
Heart failure	300 (44%)		390 (50%)	188 (59%)	<0.001
Rhythm- or conduction disorder	157 (23%)		210 (27%)	73 (23%)	0.14
Diabetes	132 (19%)		168 (22%)	81 (26%)	0.07
Hypertension	194 (28%)		257 (33%)	133 (42%)	<0.001
*Heart failure*					
Etiology of heart failure					<0.05
Ischemic origin	302 (44%)		392 (50%)	140 (44%)	
Non-ischemic origin	386 (56%)		386 (50%)	177 (56%)	
Atrial fibrillation at admission	159 (23%)		178 (23%)	49 (16%)	0.01
Left ventricular ejection fraction					<0.05
Good	199 (29%)		225 (29%)	91 (29%)	
Moderate	187 (27%)		156 (20%)	76 (24%)	
Poor	302 (44%)		396 (51%)	149 (47%)	
*Laboratory values*					
Sodium	137 ± 5		137 ± 6	135 ± 6	<0.001
Potassium	4.0 ± 0.6		4.2 ± 0.7	4.6 ± 0.9	<0.001
Urea (median, IQR)	7.2 (5.7–9.3)		10.6 (8.2–14.4)	23.5 (17.5–30.8)	<0.001
eGFR (median, IQR)	75 (66–89)		47 (39–53)	20 (14–25)	<0.001
Creatinine (median, IQR)	80 (71–96)		123 (109–142)	258 (215–346)	<0.001
Hemoglobin	8.3 ± 1.3		8.1 ± 1.4	6.9 ± 1.5	<0.001
Hyponatremia	221 (32%)		224 (29%)	151 (48%)	<0.001
Hypokalemia	106 (15%)		98 (13%)	23 (7%)	<0.001
Hyperkalemia	38 (6%)		81 (10%)	85 (27%)	<0.001
Anemia	262 (38%)		334 (43%)	244 (77%)	<0.001
*Therapy during ICCU hospitalization*					
Intubation		69 (10%)	117 (15%)	57 (18%)	0.001
Resuscitation		19 (3%)	36 (5%)	15 (5%)	0.13
Mechanical circulatory support‡		34 (5%)	41 (5%)	29 (9%)	0.02
Inotropics		196 (29%)	253 (33%)	123 (39%)	0.01
Beta-blocker		146 (21%)	111 (14%)	47 (15%)	0.001
Antiarrhythmics		115 (17%)	154 (20%)	45 (14%)	0.06
Calcium antagonist		77 (11%)	102 (13%)	72 (23%)	<0.001
Digitalis		300 (44%)	347 (45%)	87 (27%)	<0.001
ACE-inhibitor or ARB		422 (61%)	430 (55%)	113 (36%)	<0.001
Diuretics		640 (93%)	718 (92%)	257 (81%)	<0.001
Nitrates		234 (34%)	289 (37%)	121 (38%)	0.24
Nitroprusside		46 (7%)	74 (10%)	39 (12%)	0.01
Antiplatelet agents		200 (29%)	189 (24%)	71 (22%)	0.04
Oral anticoagulant		351 (51%)	406 (52%)	136 (43%)	0.02

ACE, Angiotensin-converting enzyme; ARB, Angiotensin receptor blocker; BMI, Body Mass Index; CABG, coronary artery bypass graft; eGFR, estimated glomerular filtration rate; ICCU, intensive cardiac care unit; IQR, interquartile range

*p for any difference

†Percutaneous coronary intervention and/or CABG

‡Intra-aortic balloon pump and/or left ventricular assist device and/or extracorporeal membrane oxygenation

Regarding therapy, patients with renal impairment were more frequently treated with intubation and mechanical ventilation, mechanical circulatory support and inotropic agents (Table 1). Moreover, the degree of renal impairment was associated with lower in-hospital usage of beta-blockers, ACE-inhibitors and diuretics.

### Renal function and outcome

**Fig 2:**
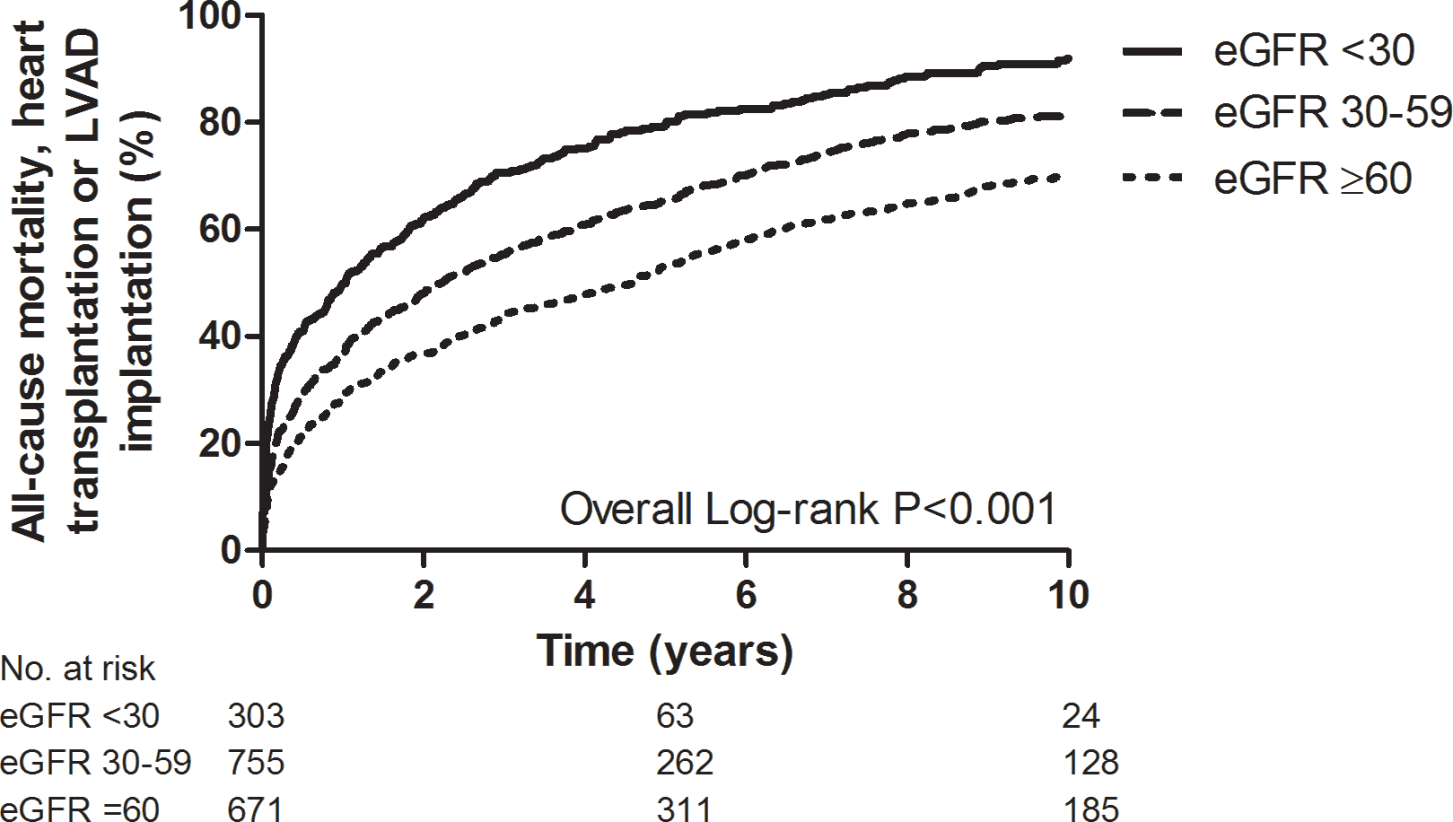
Kaplan-Meier curve of patients with acute heart failure according to the renal function. eGFR, estimated glomerular filtration rate in mL/min/1.73 m^2^; LVAD, left ventricular assist device.

The median survival of patients with a severely impaired, moderately impaired and preserved renal function was 1.0, 2.1 and 4.4 years, respectively. The impact of renal function on outcome is shown in Fig 2 and Table 2. Patients with a severely impaired renal function had the worst prognosis both at short- and long-term. These findings remained unchanged after multivariable adjustment for other prognostic factors. Although the influence of renal function on prognosis became less prominent with longer duration of follow-up, renal function still remained a strong predictor of the composite endpoint of all-cause mortality, heart transplantation and LVAD implantation.

**Table 2:** Prognosis at different follow-up moments according to renal function.

	All-cause mortality, heart transplantation or LVAD implantation	Univariable analysis*	Multivariable analysis*
*30 days*			
eGFR ≥60	10%	Reference	Reference
eGFR 30–59	14%	1.51 (1.10–2.08)	1.50 (1.06–2.11)
eGFR <30	24%	2.85 (1.99–4.08)	2.32 (1.55–3.47)
*1 year*			
eGFR ≥60	28%	Reference	Reference
eGFR 30–59	36%	1.41 (1.17–1.69)	1.34 (1.11–1.62)
eGFR <30	50%	2.21 (1.79–2.73)	1.81 (1.44–2.28)
*10 years*			
eGFR ≥60	69%	Reference	Reference
eGFR 30–59	81%	1.42 (1.33–1.51)	1.24 (1.09–1.40)
eGFR <30	92%	2.14 (1.99–2.31)	1.68 (1.43–1.96)

eGFR, estimated glomerular filtration rate in mL/min/1.73 m2; LVAD, left ventricular assist device

*Odds ratio with 95% confidence interval (CI) for 30-day outcome, hazard ratio with 95% CI for 1-year and 10-year outcome

**Fig 3:**
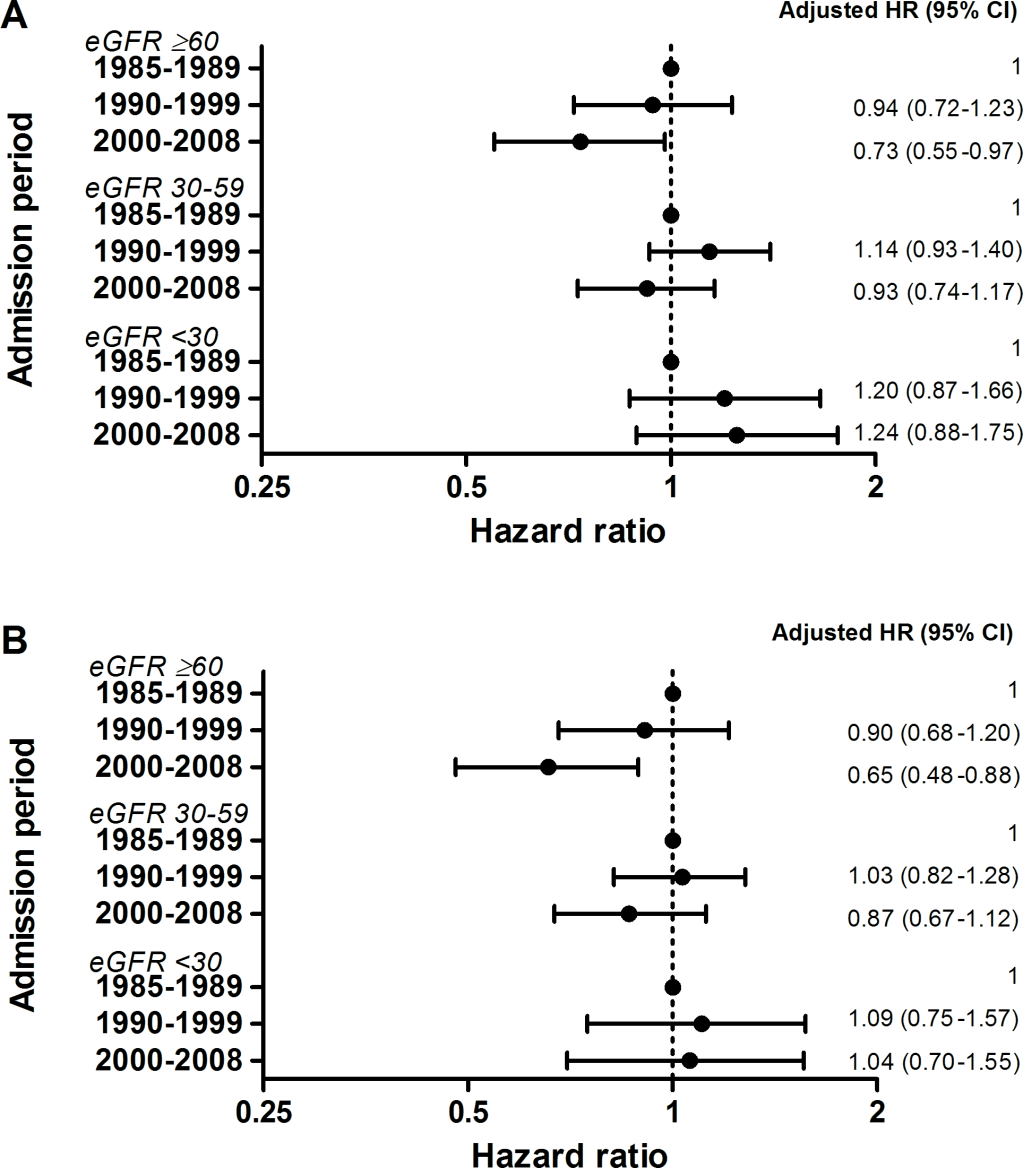
**Prognosis over time among (A) the total population and (B) the 30-day survivors of patients with acute heart failure.** Results were divided into three groups according to the renal function. CI, confidence interval; eGFR, estimated glomerular filtration rate in mL/min/1.73 m^2^; HR, hazard ratio.

Over time, the 10-year outcome of patients with a preserved renal function improved significantly, both unadjusted (HR 0.70 [95% CI 0.61–0.81] for most recent period versus first period) and after adjustment for confounding variables (adjusted HR 0.73 [95% CI 0.55–0.97]; Fig 3A). This improvement was more pronounced among the 30-day survivors (adjusted HR 0.65 [95% CI 0.48–0.88]; Fig 3B). In contrast, this pattern was not present in patients with renal dysfunction. Consequently, the prognosis of these patients did not improve over time.

### Anemia and outcome

Almost 50% of the patients were found to have anemia. The characteristics of these patients differed in some aspects from those without anemia (Table 3). Anemic patients more frequently had previous HF and atrial fibrillation at admission. Importantly, they more often had impaired renal function.

**Table 3:** Baseline characteristics and therapy of patients with and without anemia.

		Anemia +	Anemia -	p-value
No. of patients		850 (48%)	919 (52%)	
*Baseline*				
Age (mean, y)		63.1 ± 14.5	64.1 ± 15.0	0.15
Male		565 (67%)	560 (61%)	0.02
BMI		24.8 ± 4.8	25.4 ± 5.2	0.20
*Medical history*				
Myocardial infarction		336 (40%)	362 (39%)	0.95
Coronary revascularization*		199 (23%)	183 (20%)	0.07
Heart surgery (not CABG)		131 (15%)	102 (11%)	0.01
Heart transplantation		8 (0.9%)	1 (0.1%)	0.02
Waiting for heart transplantation		22 (2.6%)	12 (1.3%)	0.05
Heart failure		440 (52%)	425 (46%)	0.02
Rhythm- or conduction disorder		215 (25%)	218 (24%)	0.44
Diabetes		199 (23%)	181 (20%)	0.06
Hypertension		271 (32%)	308 (34%)	0.47
*Heart failure*				
Etiology of heart failure				>0.05
Ischemic origin		387 (45%)	438 (48%)	
Non-ischemic origin		463 (55%)	481 (52%)	
Atrial fibrillation at admission		141 (17%)	243 (26%)	<0.001
Left ventricular ejection fraction				>0.05
Good		260 (31%)	250 (27%)	
Moderate		192 (23%)	227 (25%)	
Poor		399 (47%)	442 (48%)	
*Laboratory values*				
Sodium		136 ± 6	138 ± 5	<0.001
Potassium		4.3 ± 0.8	4.1 ± 0.7	0.001
Urea (median, IQR)		12.6 (8.3–20.4)	8.4 (6.6–11.6)	<0.001
eGFR (median, IQR)		47 (26–64)	57 (43–73)	<0.001
Creatinine (median, IQR)		123 (94–200)	102 (82–130)	<0.001
Hemoglobin		6.7 ± 0.9	9.0 ± 0.8	<0.001
Hyponatremia		359 (42%)	233 (25%)	<0.001
Hypokalemia		100 (12%)	125 (14%)	0.28
Hyperkalemia		122 (14%)	85 (9%)	0.001
*Therapy during ICCU hospitalization*				
Intubation	151 (18%)		92 (10%)	<0.001
Resuscitation	36 (4%)		34 (4%)	0.56
Mechanical circulatory support†	80 (9%)		23 (3%)	<0.001
Inotropics	329 (39%)		238 (26%)	<0.001
Beta-blocker	128 (15%)		174 (19%)	0.03
Antiarrhythmics	143 (17%)		165 (18%)	0.53
Calcium antagonist	130 (15%)		123 (13%)	0.25
Digitalis	305 (36%)		419 (46%)	<0.001
ACE-inhibitor or ARB	417 (49%)		540 (59%)	<0.001
Diuretics	747 (88%)		854 (93%)	<0.001
Nitrates	295 (35%)		342 (37%)	0.27
Nitroprusside	73 (9%)		86 (9%)	0.57
Antiplatelet agents	238 (28%)		224 (24%)	0.08
Oral anticoagulant	383 (45%)		497 (54%)	<0.001

ACE, Angiotensin-converting enzyme; ARB, Angiotensin receptor blocker; BMI, Body Mass Index; CABG, coronary artery bypass graft; eGFR, estimated glomerular filtration rate; ICCU, intensive cardiac care unit; IQR, interquartile range

*Percutaneous coronary intervention and/or CABG

†Intra-aortic balloon pump and/or left ventricular assist device and/or extracorporeal membrane oxygenation

**Fig 4:**
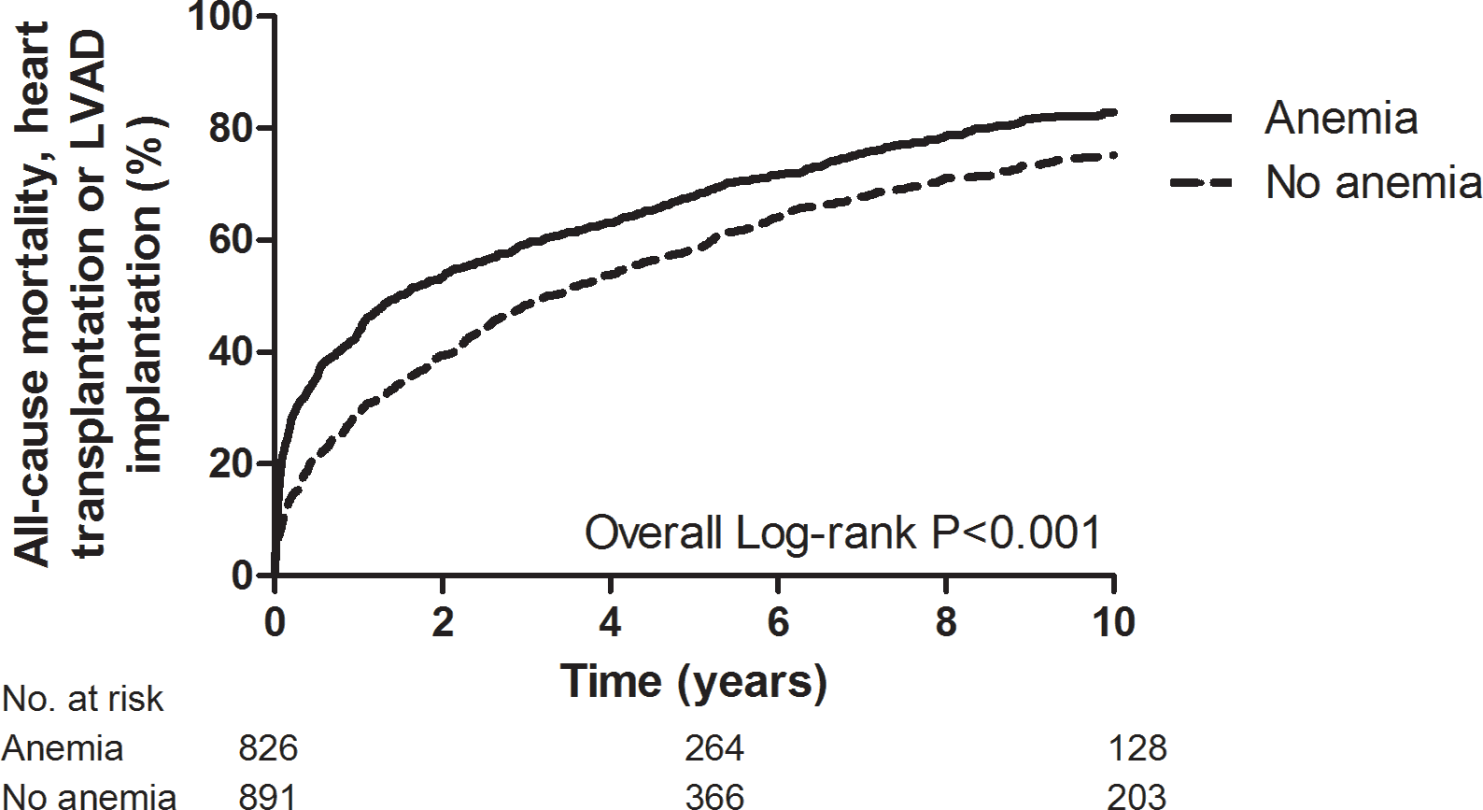
Kaplan-Meier curve of acute heart failure patients with and without anemia. LVAD, left ventricular assist device.

The prognosis of patients with anemia was worse than of patients without anemia (Fig 4). After adjustment for confounders, anemia remained significantly associated with increased for reaching the composite endpoint of all-cause mortality, heart transplantation and LVAD implantation at 30 days, 1 year and 10 years (HR 2.23 [95% CI 1.64–3.03], HR 1.58 [95% CI 1.33–1.87] and HR 1.24 [1.11–1.39], respectively; Table 4).

**Table 4:** Prognosis at different follow-up moments according to the presence of anemia.

	All-cause mortality, heart transplantation or LVAD implantation	Univariable analysis*	Multivariable analysis*
*30 days*			
No anemia	9%	Reference	Reference
Anemia	20%	2.55 (1.92–3.38)	2.23 (1.64–3.03)
*1 year*			
No anemia	28%	Reference	Reference
Anemia	43%	1.75 (1.49–2.05)	1.58 (1.33–1.87)
*10 years*			
No anemia	75%	Reference	Reference
Anemia	83%	1.35 (1.28–1.43)	1.24 (1.11–1.39)

LVAD, left ventricular assist device

* Odds ratio with 95% confidence interval (CI) for 30-day outcome, hazard ratio with 95% CI for 1-year and 10-year outcome

**Fig 5:**
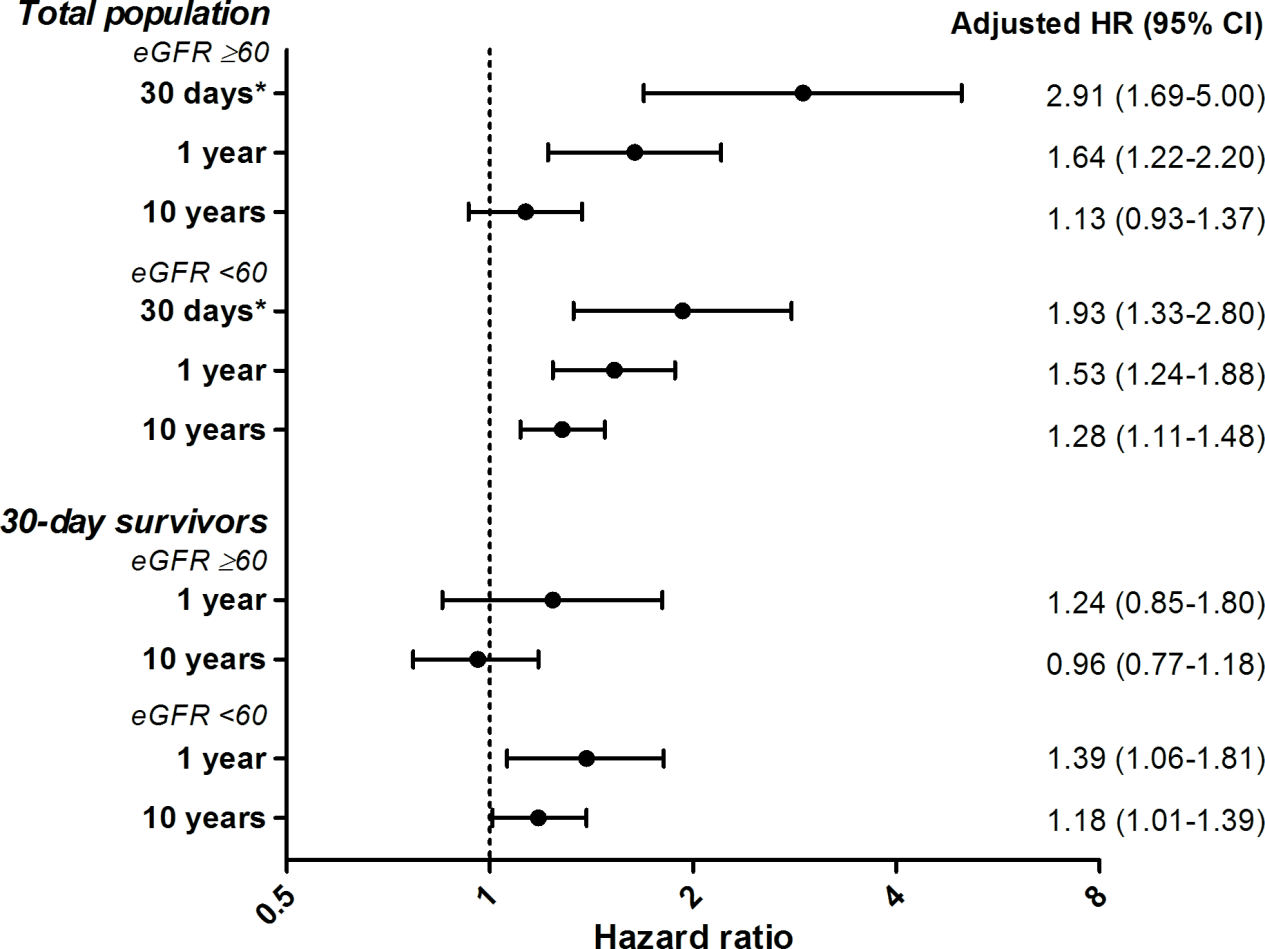
Prognostic impact of anemia at different follow-up moments in the total population and 30-day survivors. Analyses were separately done for renal impairment whether or not. CI, confidence interval; eGFR, estimated glomerular filtration rate in mL/min/1.73 m^2^; HR, hazard ratio; *outcome at 30 days was reported as odds ratio with 95% CI.

Since anemia was a predictor of poor outcome in the total population of acute HF patients, we separately analyzed whether anemia had incremental prognostic value independent from renal dysfunction (Fig 5). Among patients with a preserved renal function, anemia proved to be a strong predictor for 30-day outcome, but its prognostic value decreased with longer duration of follow-up. In contrast, anemia was associated with worse outcome both during short- and long-term follow-up among patients with renal dysfunction. This relationship persisted after the exclusion of patients who died within 30 days after admission.

## Discussion

In this prospective registry of patients with acute HF, we found that renal dysfunction was a strong predictor for poor outcome (i.e. the composite of all-cause mortality, heart transplantation and LVAD implantation) up to 10 years following initial hospitalization. Importantly, this study is the first to show that patients with acute HF and an impaired renal function had no improvement in prognosis that occurred in the last three decades. This contrasts findings in patients with a preserved renal function. Furthermore, we found that the prognostic impact of anemia was dependent on the presence of renal function. Anemia had no impact on the long-term prognosis of patients with a preserved renal function. On the other hand, anemia was associated with impaired prognosis among patients with renal dysfunction.

### Renal function and prognosis

Renal dysfunction proved to be a strong predictor of a poor outcome: the poorer the renal function, the poorer the prognosis. Among studies that demonstrated the adverse association between renal dysfunction and poor survival,[1, 2] most only used a short follow-up period, usually up to 1 year after hospitalization. Our results support and extend these findings by demonstrating that renal dysfunction continued to be a strong predictor for long-term outcome (i.e. 10 years).

It is generally assumed that the new therapeutic options for the treatment of HF developed during the last decades are responsible for the prognostic improvement in the total population of patients acute HF. Our finding that only patients with a normal renal function experienced an improved long-term prognosis in the most recently study period is novel. This contrasts with the findings currently obtained among patients with renal dysfunction. Their prognosis remained stable over time. So far, the temporal trends in prognosis have not been studied separately for patients with and without renal dysfunction. Two potential mechanisms may explain this finding. First, some of the new therapeutics, like ACE inhibitors, ARBs and MRAs, that are considered to be responsible for the prognostic improvement of patients with HF over the last decades, interact with the renal function.[6] Moreover, patients with lower eGFR were also less frequently treated with diuretics during ICCU admission. Therefore, it is plausible that patients with renal dysfunction were less frequently treated with these drugs and that, in case they were treated, the optimal dose was not achieved. Indeed, we found that ACE inhibitors were less frequently prescribed during admission in patients with renal dysfunction. Although data on medical therapy during follow-up were not included in this registry, it can be assumed that this pattern of prescription continued after discharge. Another possible explanation for the disparity in temporal trends between patients with and without renal dysfunction may be the grade of their illness. Patients with renal dysfunction had more comorbidities and were more frequently treated with intubation, mechanical circulatory support and inotropics than patients with preserved renal function. This suggests that patients with renal dysfunction were more critically ill as compared to those with a preserved renal function, and they might thus experience a more progressive course of their disease and, therefore, a poorer prognosis.

### Anemia and prognosis

The second result of our study was the finding that anemia was associated with both an impaired short- and long-term prognosis among patients with acute HF. The relation between anemia and adverse outcome in patients with acute HF has been published previously, although the data are not consistent.[10–13] Two studies that did not report anemia to be a prognosticator of poor outcome had study populations with quite different characteristics than ours.[10, 13]

When we studied the prognostic value of anemia in more detail, we found that anemia was an independent predictor of short-term outcome in all patients, irrespective of renal function. However, while anemia also was independently associated with an impaired outcome during long-term follow-up in patients with renal dysfunction, its presence had no incremental long-term prognostic impact in patients with a preserved renal function. The reasons for this difference are not totally clear. A possible explanation may be the actual cause of the anemia. However, as we were not able to assess the exact etiology of the anemia, the following hypothesis should be studied further in the future.

Anemia in patients with HF is well known, and has been attributed to multiple factors including iron deficiency, renal dysfunction, HF as a chronic disease and hemodilution.[14] The iron status was not assessed in our patients so we cannot make any conclusions as whether there was a difference in iron status between patients with and without renal dysfunction. The fact that anemia was associated with impaired long-term outcome in patients with renal dysfunction but not in patients with a preserved renal function might be due to the fact that patients with renal dysfunction more frequently had ‘true anemia’.

Hemodilution is one of the potential causes of anemia in patients with HF.[17] The causal factor in that case is a low hemoglobin level caused by an increased extracellular volume. When the extracellular volume decreases, for example by diuretic therapy, the hemoglobin level will increase and the patient will no longer be classified as having anemia. Therefore, in case of hemodilution anemia should be seen as a marker of fluid retention, just as sodium level. We hypothesize that hemodilution as the only cause of anemia was more frequent in patients without renal dysfunction than in those with renal dysfunction. Probably, patients with an impaired renal function had also anemia based on hemodilution but in addition, could also have suffered from ‘true anemia’. There are several reasons for such a phenomenon. First, it is well known that renal failure is associated with anemia.[14] Second, in our study, chronic HF was more common among patients with renal dysfunction than among those without renal dysfunction. Since chronic HF has been associated with elevated plasma levels of cytokines,[18] chronic HF can cause anemia of chronic diseases. These cytokines suppress the erythropoietic stem cells in the bone marrow and reduce the release of iron form the reticulo-endothelial system, resulting in anemia.[19]

The so-called cardiorenal anemia syndrome has not been investigated extensively in patients with acute HF. Investigators form the ATTEND registry also found anemia to be a strong predictor of in-hospital mortality both among patients with and without renal dysfunction.[20] Furthermore, their results with respect to the 1-year outcome were consistent with our data. In addition, these authors also showed that anemia had additive prognostic value for increased 1-year mortality only in the patients with renal dysfunction but not in those with a preserved renal function.[21] Because these investigators used anemia at discharge as predictor, and thus made hemodilution less likely as cause from anemia, this supports our hypothesis of ‘true anemia’ among patients with renal dysfunction. Our data provide new evidence on the very long-term prognosis of patients with acute HF since we found that anemia, even after 10 years of follow-up, continued to have additive prognostic value among patients with renal dysfunction.

### Strengths and limitations

The unique strength of our study is the duration of the follow-up of 10 years after the initial hospitalization. This enabled us to investigate the prognostic impact of renal dysfunction, anemia, as well as their interrelationship on short- en (very) long-term. Research covering three decades with such a long follow-up time is quite unique in this research field.

Despite these strengths, some limitations should be considered in the interpretation of the results of this study. Since our study was done in a tertiary referral hospital, external validity could have been affected. However, despite the fact that our hospital was a tertiary referral center, a significant part of our patients still were primary and secondary referrals. Therefore, our population consisted of patients within the whole, broad range of patients admitted with acute HF. Second, analyses were made on a composite outcome and therefore caution is needed when interpreting the estimates of the covariates, since these are estimates on the composite outcome only and not on the separate outcomes. Third, we were not able to identify the cause of anemia in all patients, nor were we always able to assess whether patients had chronic or acute renal dysfunction. Furthermore, while it has been suggested that changing hemoglobin and creatinine levels during admission may influence prognosis,[2, 22] the design of our study did not allow us to assess trends in hemoglobin and creatinine levels. Finally, since we had no data on the ethnicity of our patients, we could not multiply for black race in the MDRD formula. Therefore, the eGFR that we employed might be an underestimation of the real renal function. However, such misclassification could have only led to underestimation of the effects observed.

### Conclusions

We found renal dysfunction to be a strong predictor of both short- and long-term composite endpoint of all-cause mortality, heart transplantation and LVAD implantation among patients with acute HF. In addition, we established that the long-term prognosis of patients with a preserved renal function significantly improved over the last decades. However, in patients with renal dysfunction, the prognosis did not improve over the last decades. These findings emphasize the importance of renal dysfunction as comorbidity in patients with HF and underscore the need for new therapeutic modalities, especially for patients with renal dysfunction. Furthermore, we established anemia as a prognosticator of short-term outcome both among acute HF patients with and without renal dysfunction. Among patients with renal dysfunction, the presence of anemia was also associated with impaired long-term prognosis. Anemia did not influence the long-term prognosis of patients with preserved renal function. Further research should be undertaken to investigate the pathogenesis of the prognostic impact of anemia and renal dysfunction among patients with acute HF.

## Supporting information

S1 Dataset (SAV)
